# The Trans-Axillary Vein Approach for the Ablation of Anterior–Septal, Anterior, and Anterior–Lateral Accessory Pathways in Children: More than an Alternative to the Femoral Vein

**DOI:** 10.3390/jcm14030659

**Published:** 2025-01-21

**Authors:** Paola Ferrari, Giovanni Malanchini, Raul Limonta, Gabriele Ferrari, Cristina Leidi, Paolo De Filippo

**Affiliations:** 1 ASST Papa Giovanni XXIII Hospital, 24123 Bergamo, Italygferrari@asst-pg23.it (G.F.); pdefilippo@asst-pg23.it (P.D.F.); 2 School of Medicine and Surgery, University of Milan-Bicocca, 20100 Milan, Italy

**Keywords:** accessory pathway, children, ablation, pediatric, trans-axillary approach

## Abstract

**Background**: Catheter ablation of right anterior, anterior–lateral, and anterior–septal accessory pathways is still challenging in children, even after seminal improvements in mapping and catheter design over the last years. The trans-jugular approach was described as an alternative to the femoral vein recently. As a direct comparison between the femoral approach and the superior approach using the axillary vein was lacking, we conducted the present study. **Methods:** Twenty-two pediatric patients were enrolled in this retrospective study. Patients with prior ablation attempts were excluded. Another 22 consecutive patients with the same AP localizations were selected as a control group and treated with ablation through the femoral vein. Left axillary vein cannulation was performed advancing an 18-gauge needle using fluoroscopic landmarks (the first rib below the inferior border of the clavicle). All mapping and ablations of accessory pathways were performed with a 7 F deflectable radiofrequency ablation catheter. The main outcome of this study was ablation success at 1 year. Recurrences were defined as a relapse of preexcitation on a 12-lead electrocardiogram and/or documented supraventricular tachycardia. **Results:** There were no significant differences in sex, age, or weight between groups. No complications occurred acutely or during follow-up. There were no significant differences in acute success rates between the two groups (19/22 vs. 22/22; *p* = 0.56) at 24 h ECG recordings. At the 1-year follow-up the total recurrence rate was 15.9% (7/44 patients); there was a significantly lower recurrence rate among patients in the trans-jugular group (27.2% vs. 4.5%; *p* = 0.039). **Conclusions:** The present study suggests that the trans-axillary vein approach is a safe and effective alternative to the classical femoral approach in pediatric patients.

## 1. Introduction

Catheter ablation of right anterior, anterior–lateral, and anterior–septal accessory pathways (APs) is still challenging, even after seminal improvements in mapping and catheter design over the last years [1]. Multiple approaches (inferior or superior) are often necessary, and while both cryo- and radiofrequency (RF)-ablation were reported to be equally successful, cryoablation may be associated, but only in some reports, with a higher rate of recurrence [1,2].

Recently, the trans-jugular approach was described as an alternative approach for right-sided procedures in patients with congenital or acquired obstruction of the inferior vena cava or when aiming to improve catheter stability when ablating at the anterior aspect of the tricuspid annulus [2,3].

The axillary vein approach has been used for decades for pacemaker implantation, and is now accepted as a safe and effective approach, given the low risk for an unsuccessful procedure [4,5]. Axillary vein access is well established and is used by numerous operators as a technique for central venous catheters insertion; this procedure offers an optimal success rate and is routinely used on a daily basis by cardiac electrophysiologists both in adult and pediatric patients, allowing extreme confidence in performing this procedure during ablation [4]. This venous access may, therefore, offer an alternative safe approach to the right-sided arrhythmic substrate in children, and has been routinely used as the first choice for RF applications at our institution since 2017, due to our relevant experience in pediatric pacing procedures and its optimal safety profile, with the aim of improving catheter-to-tissue contact, a main limitation of the femoral approach when ablating on the anterior part of the tricuspid annulus [3,6]. It has been demonstrated that irreversible thermal lesion formation occurs when a temperature of about 50 °C is reached [7]. Impedance variation during RF erogation has been demonstrated to have a direct relationship with tissue contact quality: total electrode impedance variation is greater when a wider part of the electrode surface is in contact with tissue [8]. A study by Reichlin et al. demonstrated that the initial impedance drop and contact force are strictly related to one another during in vivo ablation, and that a median impedance drop within the first 10 s can be regarded as a reliable indicator of good catheter contact, which could allow high success rates for AP ablation, both acute and long-term [9].

As a direct comparison between the classical femoral approach and the superior approach using fluoroscopy-guided puncture of the superior axillary vein was lacking, we conducted this retrospective study, aiming to verify the acute success rate, recurrence rate, and complications of radiofrequency catheter ablation through a trans-axillary vein approach in the treatment of right anterior, anterior–lateral, and anterior–septal APs in our pediatric population (aged < 18 years) compared to the conventional femoral approach. We also performed a secondary endpoint analysis that aimed to evaluate whether axillary vein access facilitated better physical parameters during RF ablation compared to femoral vein access in terms of impedance drop and temperature gain.

## 2. Methods

### 2.1. Patient Population

This was a retrospective cohort study enrolling consecutive patients who underwent catheter ablation at Papa Giovanni XXIII Hospital, Bergamo, from January 2017 to January 2022. This study was approved by the Institutional Review Board and fully complied with the Declaration of Helsinki. Twenty-two patients were enrolled who met inclusion criteria: aged < 18 years and had a guideline-approved indication for ablation of a right anterior, anterior–lateral, and anterior–septal AP. Patients with prior ablation attempts were excluded. Another 22 consecutive patients with the same AP localizations were selected as a control group; these were treated with ablation via the femoral vein approach. No crossover was permitted between the two groups.

Written informed consent was given by both parents of all patients, and patients older than 12 years had the right to express their opposition to this study prior to the procedure.

### 2.2. Electrophysiological Study (EPS) and Electro-Anatomical Mapping (EAM)

Every procedure was performed under general anesthesia; antiarrhythmic therapy was discontinued for at least five half-lives before admission to hospital. Standard catheters (Inquiry™ 5 F or 6 F, Abbott, Abbott Park, IL, USA; Supreme™ 5 F or 6 F, Abbott, Abbott Park, IL, USA) were placed in the coronary sinus and in the His bundle region or in the right ventricle via left femoral vein accesses. To determine indication for catheter ablation, before and during isoproterenol infusion, programmed stimulation with incremental and rapid atrial pacing, atrial single, double, and triple premature extra stimuli, and ventricular overdrive pacing, as needed, were performed. The EnSite Precision™ system (Abbott Medical Italia SRL, Sesto San Giovanni, Milano, Italy) 3D mapping system was used in all procedures.

Pre-excitation was mapped during sinus rhythm for manifest APs, targeting the earliest V signal compared to the delta wave on the surface ECG, the presence, amplitude, and precocity of AP potential, and unipolar QS signal recording from the mapping catheter. In case of concealed APs, mapping of the earliest retrograde atrial electrogram was performed during ventricular pacing or orthodromic atrioventricular reentrant tachycardia (AVRT).

### 2.3. Trans-Axillary Approach and Ablation Procedure

Left axillary vein cannulation was performed advancing an 18-gauge needle using fluoroscopic landmarks (the lateral edge of the first rib below the inferior border of the clavicle), as previously described, after the injection of a small amount of contrast medium (5–10 mL) from the left antecubital vein [6]. Upon successful vein puncture, a guidewire was inserted and positioned in the superior vena cava. In order to favor catheter maneuverability, only short introducers were used.

**Figure 1:**
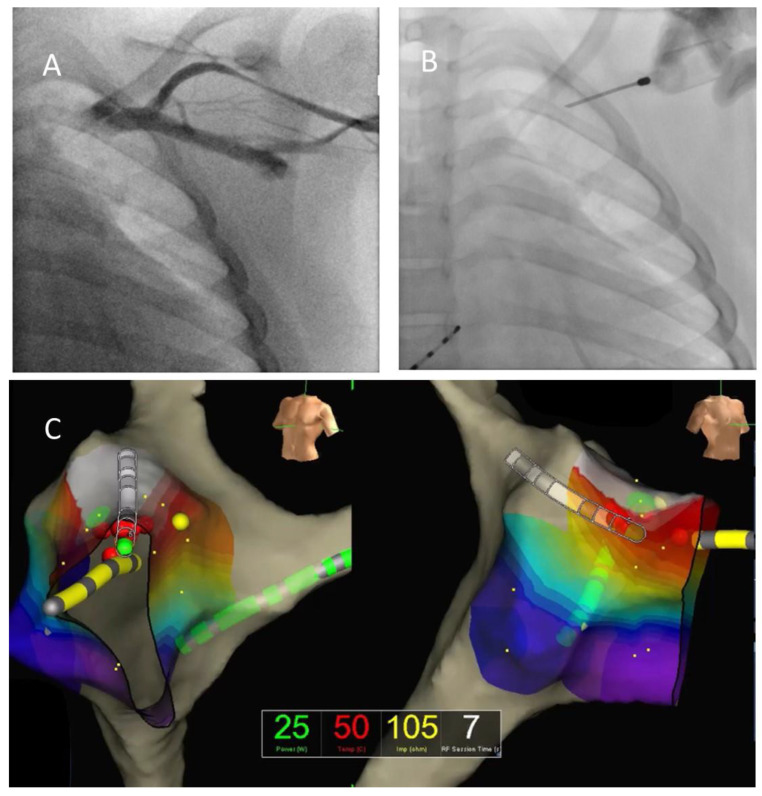
Trans-axillary ablation of anterior accessory pathway. Panel (**A**): contrast medium venography; panel (**B**): venous puncture over first rib; and panel (**C**): catheter ablation of anterior accessory pathway using mapping system to guide its localization.

All mapping and ablations were performed from a classical femoral approach in the control group and from the axillary approach in the study group, with a 7 F deflectable radiofrequency (RF) ablation catheter (Therapy™ 7 F, 4 mm tip 5 F, Abbott, Abbott Park, IL, USA). Ablations were performed in temperature-controlled mode, with a set point of 65 °C. The power was gradually up-titrated, starting from 20 W, until an adequate temperature at the catheter–tissue interface was achieved and according to the risk of damage to the AV node or other structures, as decided by the operator. If interruption of the conduction over the AP was obtained, the operator waited for 30 min. Acute success of the procedure was defined as the absence of conduction over the AP after this observation time, confirmed by the administration of adenosine. The procedure was stopped after performing a post-ablation electrophysiological study and adenosine infusion (see Figure 1).

Data on duration, power, temperature, and impedance were extracted from an automatic database generated by the navigation system for all radiofrequency (RF) applications lasting longer than 10 s. The system reported 102 values per second for each physical variable analyzed. Impedance drop after 10 s was calculated as the difference between the average impedance during the first second and the average impedance during the tenth second. Total impedance drop was calculated as the difference between the average impedance during the first second and the average impedance during the last second. The average temperature gain normalized for average power was calculated as the difference between the average temperature during the RF application and the temperature at the start of the erogation divided by the average power delivered.

### 2.4. Follow-Up

Recurrences were defined as a relapse of preexcitation on a 12-lead electrocardiogram and/or documented supraventricular tachycardia. Standard ECGs were performed 1 h and 24 h post-procedure on all patients. After discharge, patients were followed at 6 and 12 months after ablation by clinical evaluation with standard ECG and ECG Holter monitoring to assess the presence or absence of conduction over the accessory pathway.

All transient or permanent complications were noted and considered as safety endpoints. Complications were defined as any procedure-related incident requiring additional diagnostic or therapeutic measures beyond standard care.

### 2.5. Statistical Analysis

Continuous variables were expressed as mean ± standard deviation (and range of minimum and maximum values); they were tested for normal distribution with the Shapiro–Wilk test and compared with the unpaired Student’s *t*-test if normally distributed, or with the Mann–Whitney U test if not normally distributed. Categorical variables were reported as frequencies and compared with the Chi-square test or Fisher’s exact test, as appropriate. A *p* value < 0.05 was considered statistically significant. All statistical analyses were performed using Stata 13.

## 3. Results

### 3.1. Baseline and Procedural Characteristics of Patient Population and Control Group

Baseline and procedural characteristics of the patient population and the control group are summarized in Table 1.

There were no significant differences in sex, age, weight, or height between groups. Only two patients presented with structural heart diseases. There were more symptomatic patients among those treated with the trans-femoral approach. No complications occurred acutely or during follow-up.

### 3.2. Acute and Long-Term Results

**Figure 2:**
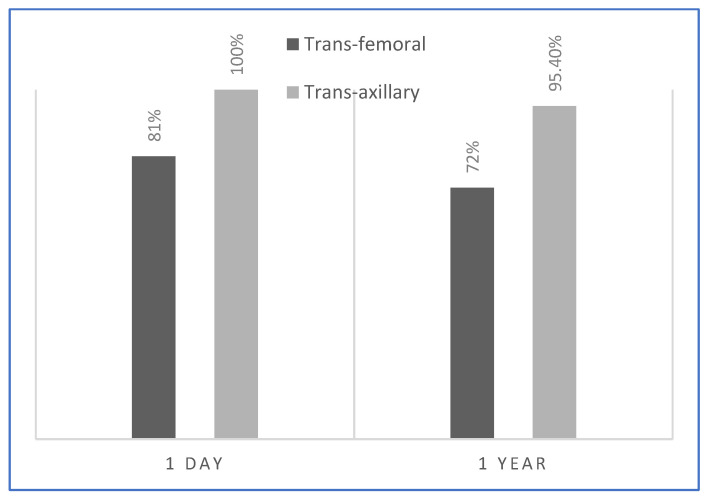
Acute and long-term success rates of anterior, anterior–septal, and anterior–lateral accessory pathways—femoral vs. axillary approaches.

There were no significant differences in acute success rates between the two groups (19/22 vs. 22/22; *p* = 0.56) at the 24 h ECG recording. At the 1-year follow-up the total recurrence rate was 15.9% (7/44 patients); there was a significantly lower recurrence rate among those in the trans-jugular group (27.2% vs. 4.5%; *p* = 0.039) (see Figure 2).

### 3.3. Ablation Quality and Bio-Physics

A total of 153 radiofrequency erogation episodes were performed among 44 patients; 90 axillary vein (axillary vein radio-frequency—AVRF) and 63 femoral vein (femoral vein radio-frequency—FVRF) applications were analyzed in this study. FVRFs were on average shorter than AVRFs (22 ± 18 s vs. 39 ± 33 s; *p* < 0.01), but the mean average powers delivered were similar between the two groups (25 ± 5 W for AVRF vs. 25 ± 6 W for FVRF; *p* = 0.76). AVRF showed better performance in terms of average temperature during RF applications and maximum temperature reached (47 ± 3 °C vs. 44 ± 2 °C, 51 ± 4 °C vs. 48 ± 3 °C, and 10 ± 3 °C vs. 7 ± 2 °C, respectively; all *p* values were < 0.01). The average temperature gain normalized for the average power delivered was superior for AVRF compared to FVRF (0.42 ± 0.14 °C/W vs. 0.31 ± 0.13 °C/W; *p* < 0.01). The impedance drop at 10 s and the total impedance drop were significantly higher for AVRF than they were for FVRF, considering both absolute values and percentages (5.7 ± 3.6 Ω vs. 3.5 ± 4.1 Ω corresponding to 5.5 ± 3.2% vs. 3.1 ± 3.7%, and 6.4 ± 5.3 Ω vs. 2.6 ± 7.1 Ω corresponding to 6.2 ± 5.0% vs. 2.4 ± 6.3, respectively; all *p* values were < 0.01) (see Table 2).

### 3.4. Radiological Exposure

The mean fluoroscopy time in this study was 49 ± 42 s, the mean dose–area product (DAP) and the mean radiation dose were 5.6 ± 4.4 µGm^2^ and 0.45 ± 0.35 mGy, respectively, and the mean procedural duration was 185 ± 55 min (Table 3). The fluoroscopy time was higher in patients treated in the trans-axillary vein access group compared to those treated using historical femoral vein access, but the difference was not statistically significant (53 ± 38 s vs. 35 ± 51 s; *p* = 0.09). The DAP and radiation dose were higher for the study group than for the control group (6.5 ± 3.2 µGm^2^ vs. 3.6 ± 5.2 µGm^2^; *p* = 0.02, and 0.52 ± 0.26 mGy vs. 0.28 ± 0.40 mGy; *p* = 0.03, respectively).

## 4. Discussion

**Figure 3:**
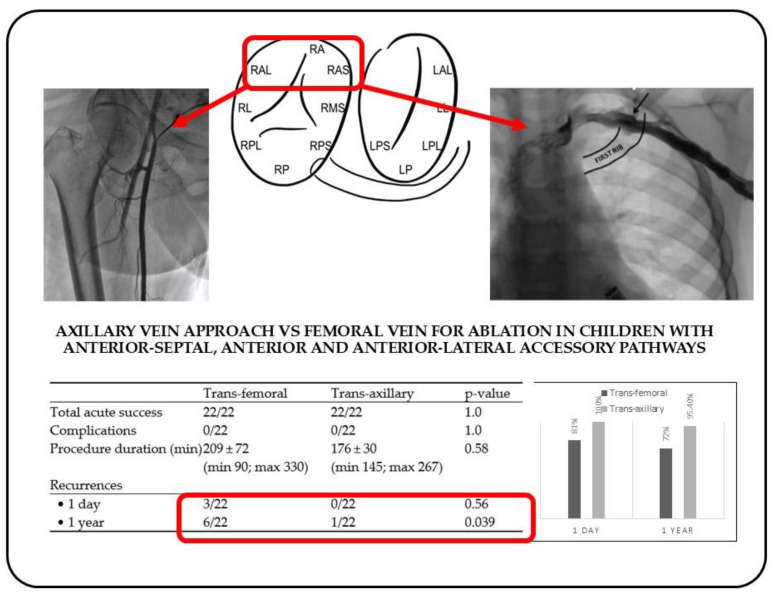
Summary of study findings: axillary vein approach led to lower long-term recurrence rate in anterior–lateral, anterior, and anterior–septal accessory pathway ablation.

In the present study, we proposed a trans-axillary vein approach, compared to the standard femoral approach, for radiofrequency catheter ablation of right-sided accessory pathways (APs) in pediatric patients. Our findings show comparable acute success rates between the two groups (100%), no procedural complications, and a lower, statistically significant recurrence rate in the trans-axillary group (4.5% vs. 27.2%; *p* = 0.039). These findings underscore the potential of the trans-axillary approach as a valuable alternative, particularly in pediatric cases, for ablation involving anterior, anterior–septal, and anterior–lateral accessory pathways (see Figure 3).

Our study, showing that this method is not only safe, but also reduces recurrence rates compared to conventional methods, is consistent with previous findings, mainly regarding the trans-jugular approach [1,3]. Kovach et al. highlighted that pediatric ablation of anterior–septal and mid-septal APs achieved acute success rates exceeding 95%, although recurrence rates could reach 25% after one year [1]. Similarly, Drago et al. reported significantly lower recurrence rates (4%) for the trans-jugular approach compared to the femoral approach (38%) during cryoablation of right-sided APs [3]. The trans-axillary approach offers unique procedural advantages. Silvetti et al. demonstrated that this method also results in fewer complications in other settings compared to the subclavian approach, a critical factor during procedures in very young patients [5]. Stability, which reduces incomplete ablations, has been underscored by numerous studies that showed that contact force is directly related to ablation outcomes [10]. Some other previous studies, also by our group, detailed a fluoroscopic-guided axillary vein puncture technique, emphasizing its safety and effectiveness in device implantation procedures [6,11]. The reason to perform this type of venous approach at our hospital was mainly the fact that operators wanted to perform ablation, especially in smaller children, utilizing a routinely performed vascular access, aiming to minimize complications. The choice of left vs. right axillary vein was mainly driven by the steep angle between the right subclavian vein and the superior vena cava, which often results in poor catheter maneuverability.

Ergül et al. confirmed these findings, reporting improved success rates for ablations performed via superior access, particularly in anatomically challenging cases [2].

The analysis of bio-physical parameters registered during RF applications revealed that axillary vein access facilitated higher average and maximum temperatures. Applications performed via the axillary vein reached a mean temperature significantly higher than those through the femoral vein, despite the increase in power delivery. A much more stable catheter–tissue contact was inferable by higher values of average temperature gain corrected for delivered power obtained with axillary vein RF applications.

Safety is a paramount consideration, particularly in pediatric populations; therefore, specific indications and weight limitations are recommended [12]. In this study, no acute or long-term complications were observed in either group, especially regarding atrioventricular blocks and vascular complications. These findings are consistent with previous research on trans-jugular approaches, which have demonstrated low complication rates in pediatric populations [2,3]. The use of short introducers and careful anatomical assessment likely contributed to the favorable safety profile of the trans-axillary approach. The recurrence rates observed in our study merit further discussion. While the femoral approach achieved comparable acute success, its higher recurrence rate (27.2%) may be attributed to suboptimal catheter stability in the anterior regions of the tricuspid annulus. Ergül et al. reported similar findings, emphasizing that superior venous access routes, such as the axillary and jugular veins, provide more stable catheter positioning, reducing the likelihood of incomplete ablations [2]. This is particularly relevant in cases involving pediatric patients, in which anatomical constraints and smaller vascular structures impose some additional challenges. Stability during ablation likely contributes to long-term efficacy, as indirectly demonstrated by bio-physics analysis, as supported by previous study who demonstrated that catheter position influences lesion durability [13]. Moreover, the radiational exposure was not significantly higher in the study group.

Recent advancements in mapping technologies have further enhanced ablation outcomes, particularly in complex AP ablations [14]. In the last decades, comparative studies have highlighted the benefits of radiofrequency over cryoablation for pediatric ablations. Comparisons between radiofrequency and cryoablation further underscore the advantages of the trans-axillary approach. Although cryoablation offers a safer profile near critical structures, it is sometimes associated with higher recurrence rates due to lower lesion durability [2,13,14,15]. Our study suggests that radiofrequency ablation, particularly when performed with stable catheter positioning, as facilitated by the trans-axillary approach, provides very good and consistent long-term outcomes.

Alternative venous access routes, such as the jugular or axillary vein, are critical for patients with challenging anatomy. Emmel et al. demonstrated that these superior routes are invaluable in patients with inferior vena cava abnormalities [16]. Our findings support this, showcasing the trans-axillary approach as a robust alternative for pediatric patients, with the possible advantage of being an approach already in use for other implanting procedures.

Despite its benefits, we also have to underscore that the trans-axillary approach may also exhibit some limitations. The learning curve associated with this technique may initially increase procedural times and radiation exposure. Future studies should explore the impact of operator experience on outcomes and evaluate strategies to streamline the procedure, for instance, the integration of zero-fluoroscopy techniques using, for example, ultrasound-guided venous puncture [11].

Clinical and practical implications of our study extend into the field of pediatric electrophysiology: the proposed trans-axillary approach represents a shift toward individualized procedural strategies that account for anatomical and clinical complexities, and may offer some advantages at an operator-based level. Its adoption could reduce the need for multiple ablation attempts, with the aim of increasing the effectiveness of ablation lesions, improving both patient outcomes and perceived quality. Many studies highlight the advantages of alternative venous access in pediatric patients, even though there are differences in procedural techniques and outcomes. In our study, we proposed a trans-axillary approach, which was demonstrated to be more effective than the femoral approach, as it may offer similar stability. Its superior bio-physical performance combined with its favorable safety profile, together with the fact that most physicians who perform pacing procedures are also confident with this approach, are relevant contributions to its value. Both jugular and axillary approaches underscore the potential of superior venous routes to improve procedural stability and outcomes in challenging pediatric AP ablations. Further studies with larger cohorts and extended follow-up will be critical to determine if one approach or the use of radiofrequency versus cryoablation consistently yields better outcomes.

## 5. Conclusions

Our study suggests that the trans-axillary vein approach is a safe and effective alternative to the classical femoral approach for radiofrequency catheter ablation of right-sided anterior, anterior–lateral, and anterior–septal accessory pathways in pediatric patients. While both methods achieved high acute success rates without complications, the long-term success of trans-axillary ablation was demonstrated to have reduced late recurrence rates.

## Figures and Tables

**Table 1:** Baseline and procedural characteristics of patients treated in two study groups.

	Trans-Femoral	Trans-Axillary	*p*-Value
Male	14/22	16/22	0.7
Age (years)	11 ± 3 (min 4; max 16)	11 ± 3 (min 4; max 17)	0.96
Weight (kgs)	39 ± 16 (min 15; max 72)	42 ± 17 (min 20; max 100)	0.75
Height (cms)	142 ± 21 (min 96; max 180)	142 ± 16 (min 100; max 180)	0.84
Structurally normal heart	21/22	21/22	1.0
Symptomatic AVRT	17/22	9/22	0.03
Total acute success	22/22	22/22	1.0
Complications	0/22	0/22	1.0
Procedure duration (min)	209 ± 72 (min 90; max 330)	176 ± 30 (min 145; max 267)	0.58
Recurrences			
• 1 day	3/22	0/22	0.56
• 1 year	6/22	1/22	0.039

Abbreviations: AVRT, atrioventricular reentrant tachycardia.

**Table 2:** Physical parameters of 153 RF applications in two study groups.

	Trans-Axillary Access	Trans-Femoral Access	*p*-Value
Number	90 (59%)	63 (41%)	
Duration (s)	39 ± 33	22 ± 18	<0.001
Average power (W)	25 ± 5	25 ± 6	0.76
Average temperature (°C)	47 ± 3	44 ± 2	<0.001
Maximum temperature (°C)	51 ± 4	48 ± 3	<0.001
Average temperature gain/average power (°C/W)	0.42 ± 0.14	0.31 ± 0.13	<0.001
Impedance drop at 10 s (Ω)	5.7 ± 3.6	3.5 ± 4.1	<0.001
Impedance drop at 10 s (%)	5.5 ± 3.2	3.1 ± 3.7	<0.001
Final impedance drop (Ω)	6.4 ± 5.3	2.6 ± 4.7	<0.001
Final impedance drop (%)	6.2 ± 4.5	2.4 ± 6.3	<0.001

**Table 3:** Radiological data during procedures in two groups.

	Trans-Axillary	Trans-Femoral	*p*-Value
Fluoroscopy time (s)	53 ± 38	35 ± 51	0.09
DAP (μGym^2^)	6.5 ± 3.2	3.6 ± 5.2	0.02
Radiation dose (mGy)	0.52 ± 0.26	0.28 ± 0.40	0.03

## Data Availability

Data presented in this study are available on request from the corresponding author.
